# Menopause modified the association of blood pressure with osteoporosis among gender: a large-scale cross-sectional study

**DOI:** 10.3389/fpubh.2024.1383349

**Published:** 2024-05-02

**Authors:** Haidong Jin, Hongfei Zhao, Sufan Jin, Xianhong Yi, Xiaotian Liu, Chongjian Wang, Gongyuan Zhang, Jun Pan

**Affiliations:** ^1^Department of Orthopaedic Surgery, The Second Clinical Medical School, The Second Affiliated Hospital and Yuying Children's Hospital of Wenzhou Medical University, Wenzhou, Zhejiang, China; ^2^Department of Epidemiology and Biostatistics, College of Public Health, Zhengzhou University, Zhengzhou, Henan, China; ^3^Faculty Development Center (Education Supervision and Teaching Evaluation Center), Wenzhou Medical University, Wenzhou, Zhejiang, China

**Keywords:** osteoporosis, blood pressure, rural health, gender-based differences, menopause

## Abstract

**Purpose:**

This study aimed to assess the potential association between blood pressure and osteoporosis in a rural population with limited resources. Existing evidence on this association is limited, particularly in such settings.

**Methods:**

Data from 7,689 participants in the Henan Rural Cohort study were analyzed. Four blood pressure indicators [systolic blood pressure (SBP), diastolic blood pressure (DBP), mean arterial pressure (MAP), and pulse pressure (PP)] were measured. The logistic regression model and restricted cubic spline plots were used to assess the relationship between blood pressure indicators and osteoporosis prevalence.

**Results:**

Positive trends were noted between blood pressure indicators and osteoporosis prevalence in the entire group and women (*P*_trend_ < 0.05 for SBP, MAP, and PP). Women with higher SBP and PP exhibited elevated odds of osteoporosis compared with those with the lowest SBP and PP (*OR*s ranging from 1.15 to 1.5 for SBP and 1.06 to 1.83 for PP). No such associations were found in men. These relationships were only evident in postmenopausal women. Dose–response analysis confirmed these findings. Excluding participants taking hypertension medication did not alter the results.

**Conclusion:**

In resource-limited settings, higher SBP and PP are associated with the increased prevalence of osteoporosis in women, potentially influenced by menopause-related factors. This indicates that potential gender-based differences and social inequalities may affect bone health.

**Clinical trial registration:**

The Henan Rural Cohort Study has been registered at the Chinese Clinical Trial Register (Registration number: ChiCTR-OOC-15006699) http://www.chictr.org.cn/showproj.aspx?proj=11375.

## Introduction

Osteoporosis is a medical condition that causes a reduction in bone mass and deterioration of bone structure. This can lead to weakened bones that are more susceptible to fractures (1). In China, there has been a recent increase in the prevalence of osteoporosis, with rates reaching 13.5% in men and 29.0% in women (2). This increase is largely attributable to the aging population (3). It is estimated that between 60 and 120 million individuals will suffer from osteoporosis, resulting in ~5.99 million fractures and an estimated cost of 25.43 billion dollars in 2050 (4, 5). This unexpected cost can place a significant economic burden on both society and families, particularly in rural areas where medical resources are limited (3).

Elevated blood pressure, a major risk factor for death and disability globally (6), accounts for 10.4 million deaths worldwide (7). Recent research has concentrated on the relationship between osteoporosis and blood pressure as they share common regulatory mechanisms (8). Several epidemiological studies have demonstrated that patients with hypertension are at an increased risk of bone loss and decreased bone turnover (9, 10). A cross-sectional study of 270 postmenopausal Turkish women indicated that hypertension was associated with low bone mineral density (BMD), while a meta-analysis of 1,43,0431 participants and 148,048 osteoporotic fracture cases revealed a higher risk of fractures among patients with hypertension (11). However, this association may be gender-based. A longitudinal study revealed that hypertension was associated with high femoral neck BMD in men and low BMD in women (12). In addition, the previous meta-analysis concluded a similar result, in which the association between hypertension and osteoporosis was more remarkable in women than in men (11).

Although numerous studies have yielded comprehensive results on the relationship between hypertension and osteoporosis, evidence in areas with limited resources remains scarce. Furthermore, the research on the association between specific blood pressure indicators, such as systolic blood pressure (SBP), diastolic blood pressure (DBP), mean arterial pressure (MAP), and pulse pressure (PP), and osteoporosis is also limited. As a result, this study aimed to indicate whether there would be gender-based variations in the association between blood pressure indicators and osteoporosis prevalence in areas with limited resources.

## Methods

### Study participants

This research used data from the Henan Rural Cohort study (ChiCTR-OOC-15006699), previously described in detail (13). Briefly, the cohort recruited 39,259 adults aged 18–79 years residing in five rural Henan counties (Suiping, Yuzhou, Xinxiang, Tongxu, and Yima) between 2015 and 2017. The study aimed to investigate the prevalence of chronic non-communicable diseases (NCDs) like hypertension, stroke, and osteoporosis in this rural population and to explore potential links between environmental exposures, genetics, and NCD risk. Eligibility criteria included the following: subjects were permanent residents, were healthier so as to answer our questionnaire, and did not move out in the following-up.

Among 39,259 individuals who participated in the Henan Rural Cohort study baseline, only 8,475 underwent bone mineral density (BMD) testing. To ensure data quality and minimize confounding factors, we excluded participants with missing BMD data (*n* = 442) and missing blood pressure measurements (*n* = 304). Additionally, to avoid potential disruptions in bone metabolism due to chronic health conditions, we excluded individuals with a history of stroke, cancer, or kidney failure (*n* = 40). This resulted in a final analysis sample of 7,689 participants. The study protocol was approved by the Zhengzhou University Life Science Ethics Committee [Code: (2015) MEC (S128)], and all participants provided informed consent.

### Covariate measurement

Sociodemographic and lifestyle data**:** trained researchers collected data through a structured questionnaire on participants' age (categorized as 18–44, 45–64, and 65+), gender, education level (elementary or below, junior high school, and high school or above), marital status (married/cohabiting, unmarried/divorced/widowed), and average monthly income (< ¥500, ¥500-¥1000, and >¥1000). Lifestyle habits: smoking and drinking habits were categorized as never, former, or current. Physical activity measured by metabolic equivalent (MET) hours per week was classified into low, moderate, and high. More vegetable and fruit intake was defined as one person who ate vegetables and fruits over 500 g/day on average while a high-fat diet was defined as consuming more than 75 g of fat/day on average. The body mass index (BMI) was calculated from the height and weight measured using standardized equipment. Medical information: menopause status was determined through the a questionnaire. Antihypertensive medication use was defined as taking antihypertensive drugs in the past 2 weeks.

### Measurement of blood pressure and definition of hypertension

BP was measured using electronic sphygmomanometers (Omron HEM-7071A) on the right arm. After resting for at least 5 min in a seated position with their arm at heart level, participants had their BP measured three times (14, 15). The average of these three readings was used for analysis. The mean arterial pressure (MAP) and pulse pressure (PP) were calculated as follows:

MAP: 2/3 DBP + 1/3 SBP; PP: SBP – DBP

Hypertension was defined as

- Mean SBP ≥ 140 mmHg or DBP ≥ 90 mmHg
- Self-reported history of hypertension
- Taking antihypertensive medication in the past 2 weeks

### Definition of osteoporosis

The BMD of the participants' heels was measured by trained staff blinded to the study, using a Sahara clinical bone densitometer. Participants were positioned comfortably seated with specific leg angles for optimal measurement. Three measurements were taken on the left heel, or the other heel if a previous fracture was present (16). Osteoporosis was diagnosed using the WHO criteria based on T-scores calculated from the BMD measurements. The T score represents the number of standard deviations for which a person's BMD deviates from the average healthy adult population. Individuals with a T-score ≤ −2.5 were classified as having osteoporosis (17).

### Statistical analyses

Considering that a potential gender-difference association between osteoporosis and blood pressure may exist, all analyses in this research were presented by gender. Continuous variables were expressed as mean ± SD, and categorical variables were expressed as counts with percentages. Gender-difference characteristics were detected by using one-way ANOVA or Pearson's chi-square test. Blood pressure indicators were divided into five groups, and the group with the lowest levels was set as the reference group. The prevalence of osteoporosis was presented by gender and blood pressure group, and unadjusted binary logistic regression was employed to detect the trend. With adjustment of age, gender, educational level, marital status, average monthly income, smoking, drinking status activity, high-fat diet, more vegetable and fruit intake, and BMI, the binary logistic regression model was used to explore the associations between blood pressure and osteoporosis, and to test the robustness of our findings, we further explored the relationship between blood pressure indicators and the risk of osteoporosis in the older adults by gender (≥50 years old). In addition, the restricted cubic spline interpolation plot was used to explore the trend. Considering menopause status is usually related to osteoporosis (18), a stratified analysis among women by menopause status was also conducted. To test the robustness of our findings, some sensitivity analyses excluding medications for hypertension were done. All analyses were conducted via R software version 4.0.3. All tests were two-tailed, and *P* < 0.05 was regarded as statistically significant.

## Results

### Baseline characteristics of participants

This research included 2,879 men (37.44%) and 4,810 women (62.56%). Osteoporosis was identified in 405 (14.07%) men and 1,089 (22.64%) women. Women were found to be older, have lower educational levels, higher average monthly income, and were less likely to smoke or drink, less physically active, with a lower rate of a high-fat diet, but had a higher intake of vegetables and fruits and a higher BMI compared to men (all *P* < 0.05). Moreover, women had lower systolic blood pressure (121.72 ± 18.94 vs. 122.64 ± 17.58 mmHg), diastolic blood pressure (74.97 ± 10.86 vs. 76.52 ± 11.32 mmHg), and mean arterial pressure (90.56 ± 12.78 vs. 91.89 ± 12.73 mmHg), but higher pulse pressure (46.75 ± 12.53 vs. 46.12 ± 10.89 mmHg) than men (all *P* < 0.05). See Table 1 for more details. In addition, the baseline characteristics of participants by osteoporosis and hypertension are shown in Supplementary Tables 1, 2.

**Table 1:** Baseline characteristics of participants by gender.

**Variables**	**Total**	**Men**	**Women**	***P***
	***N** =* **7,689**	***N** =* **2,879**	***N** =* **4,810**	
Age, *n* (%)				< 0.001
18-	1,137 (14.79)	365 (12.68)	772 (16.05)	
45-	4,889 (63.58)	1,766 (61.34)	3,123 (64.93)	
65-	1,663 (21.63)	748 (25.98)	915 (19.02)	
Educational level, *n* (%)				< 0.001
Elementary school or below	3,377 (43.92)	949 (32.96)	2,428 (50.48)	
Junior high school	3,181 (41.37)	1,348 (46.82)	1,833 (38.11)	
High school or above	1,131 (14.71)	582 (20.22)	549 (11.41)	
Marital status, *n* (%)				0.138
Married/cohabitating	7,011 (91.18)	2,643 (91.80)	4,368 (90.81)	
Unmarried/divorced /widowed	678 (8.82)	236 (8.20)	442 (9.19)	
Average monthly income, *n* (%)			
< 500 RMB	2,444 (31.79)	985 (34.21)	1,459 (30.33)	
500–1000 RMB	2,323 (30.21)	854 (29.66)	1,469 (30.54)	
≥1000 RMB	2,922 (38.00)	1,040 (36.12)	1,882 (39.13)	
Smoking status, *n* (%)				< 0.001
Never	5,689 (74.90)	932 (32.99)	4,757 (99.73)	
Former	364 (4.79)	358 (12.67)	6 (0.13)	
Current	1,542 (20.30)	1,535 (54.34)	7 (0.15)	
Drinking status, *n* (%)				< 0.001
Never	5,918 (77.90)	1,303 (46.12)	4,615 (96.71)	
Former	1,077 (14.18)	951 (33.66)	126 (2.64)	
Current	602 (7.92)	571 (20.21)	31 (0.65)	
Physical activity, *n* (%)				< 0.001
Low	2,151 (27.98)	917 (31.85)	1,234 (25.65)	
Moderate	2,961 (38.51)	819 (28.45)	2,142 (44.53)	
High	2,577 (33.52)	1,143 (39.70)	1,434 (29.81)	
High-fat diet, *n* (%)	1,552 (20.18)	783 (27.20)	769 (15.99)	< 0.001
More vegetable and fruit intake, *n* (%)	4,060 (52.80)	1,571 (54.57)	2,489 (51.75)	0.016
Hypertension, *n* (%)	1,932 (25.13)	694 (24.11)	1,238 (25.74)	0.110
Osteoporosis, *n* (%)	14,94 (19.43)	405(14.07)	1,089 (22.64)	< 0.001
Postmenopausal women, *n* (%)	2,687 (34.95)	-	2,687 (55.86)	-
BMI (kg/m^2^, mean ± SD)	24.65 ± 3.42	24.39 ± 3.37	24.80 ± 3.44	< 0.001
SBP (mmHg, mean ± SD)	122.07 ± 18.44	122.64 ± 17.58	121.72 ± 18.94	0.034
DBP (mmHg, mean ± SD)	75.55 ± 11.06	76.52 ± 11.32	74.97 ± 10.86	< 0.001
MAP (mmHg, mean ± SD)	91.06 ± 12.78	91.89 ± 12.73	90.56 ± 12.78	< 0.001
PP (mmHg, mean ± SD)	46.51 ± 11.95	46.12 ± 10.89	46.75 ± 12.53	0.027

BMI, body mass index; SBP, systolic blood pressure; DBP, diastolic blood pressure; MAP, mean arterial pressure; PP, pulse pressure.

### Prevalence of osteoporosis in groups with different blood pressure indicators

**Figure 1:**
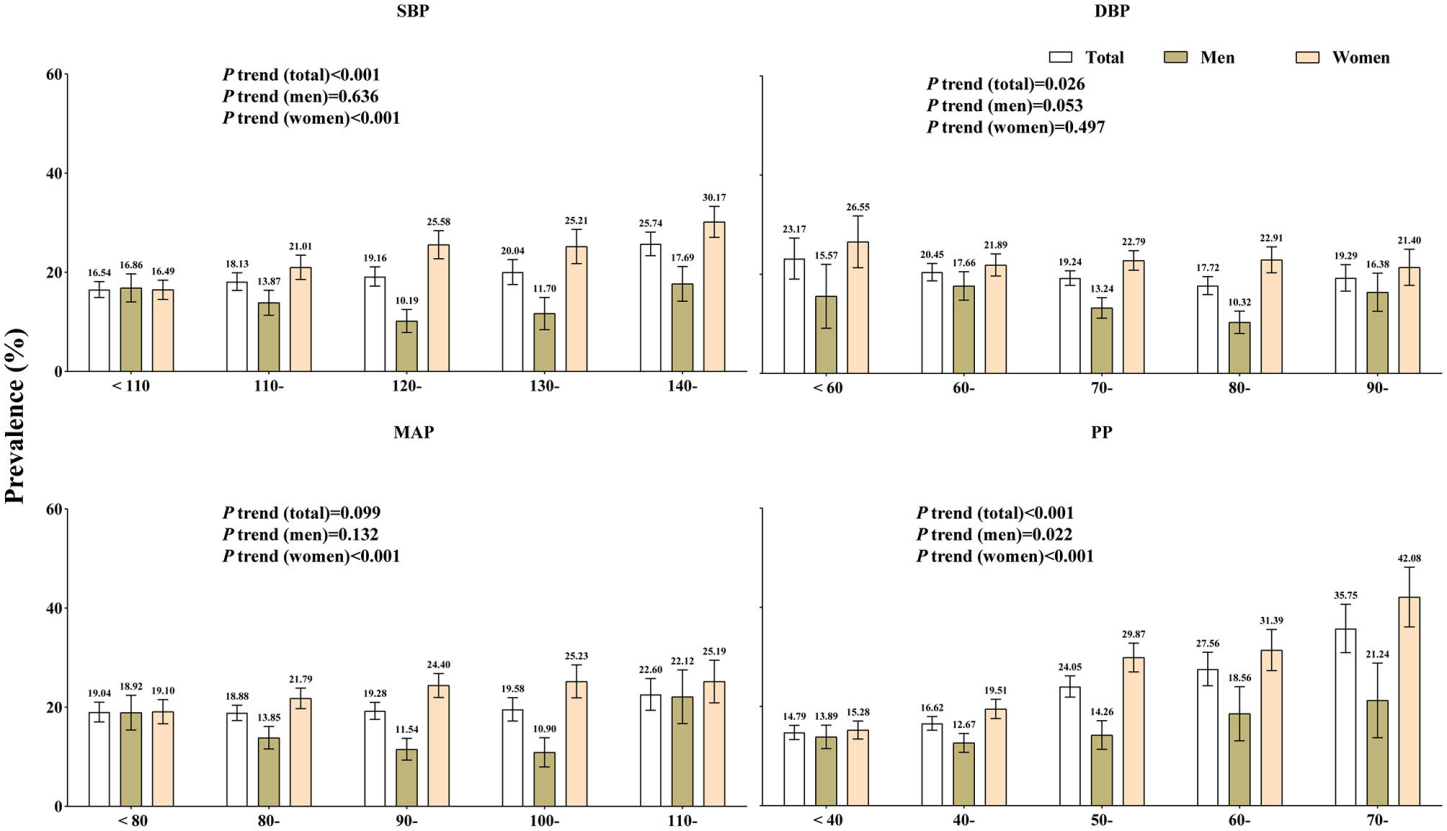
Prevalence of osteoporosis in the different blood pressure indicator groups by gender. SBP, systolic blood pressure; DBP, diastolic blood pressure; MAP, mean arterial pressure; PP, pulse pressure.

The graph in Figure 1 shows the changes in osteoporosis prevalence in different blood pressure indicator groups. In the SBP subgroups, the prevalence of osteoporosis among women increased from 16.49% (95% CI: 14.54% and 18.44%) in the group with < 110 mmHg to 30.17% (95% CI: 27.05% and 33.29%) in the 140-mmHg group (*P*_trend_ < 0.001). However, this trend was not observed in men, who had prevalence rates ranging from 16.87% to 17.67% (P_trend_ = 0.636). In MAP subgroups, the prevalence of osteoporosis among women increased from 19.10% (95% CI: 16.66%−21.54%) in the group with < 80 mmHg to 25.19% (95% CI: 20.88%−29.51%) in the 110-mmHg group (*P*_trend_ < 0.001). Again, this trend was not observed in men, who had prevalence rates ranging from 18.92% to 22.12% (*P*_trend_ = 0.132). The prevalence of osteoporosis in women increased sharply with increasing PP, from 15.28% in the group with < 40 mmHg to 42.08% in the 70-mmHg group (*P*_trend_ < 0.001). However, this trend was not observed in men. The trends remained stable even when medications were excluded from the analysis (see Supplementary Figure 1).

### Gender-difference association between blood pressure and osteoporosis

**Figure 2:**
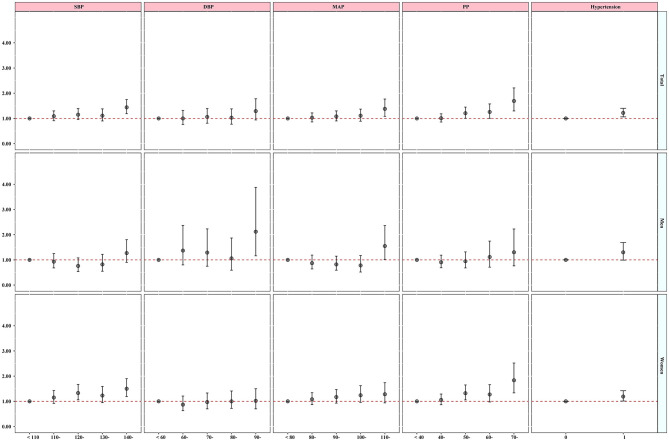
Gender-difference association on blood pressure indicators and hypertension with osteoporosis. The model was adjusted by age, gender (Total), educational level, marital status, average monthly income, smoking, drinking status, physical activity, high-fat diet, increased vegetable and fruit intake, and BMI.

As depicted in Figure 2, among women, the odds ratios (OR) and their 95% confidence intervals (CI) for four categories were as follows: 1.15 (0.92, 1.43), 1.33 (1.06, 1.67), 1.23 (0.95, 1.59), and 1.50 (1.19, 1.90) for systolic blood pressure (SBP) and 1.06 (0.87, 1.29), 1.32 (1.06, 1.65), 1.27 (0.97, 1.66), and 1.83 (1.34, 2.52) for pulse pressure (PP) compared with the reference group. However, these positive associations between blood pressure indicators and osteoporosis were significantly observed only in diastolic blood pressure (DBP) among men. Nonetheless, the dose–response relationships were only found in women, as shown in Supplementary Figure 2. These results were consistent even after excluding the medication use, as demonstrated in Supplementary Figures 3, 4. Furthermore, we also explored the relationship between blood pressure indicators and the risk of osteoporosis in the older adults by gender (≥50 years old). The results are shown in Supplementary Tables 3, 4. The results were similar to those of the main analysis.

### Sensitivity analyses

**Figure 3:**
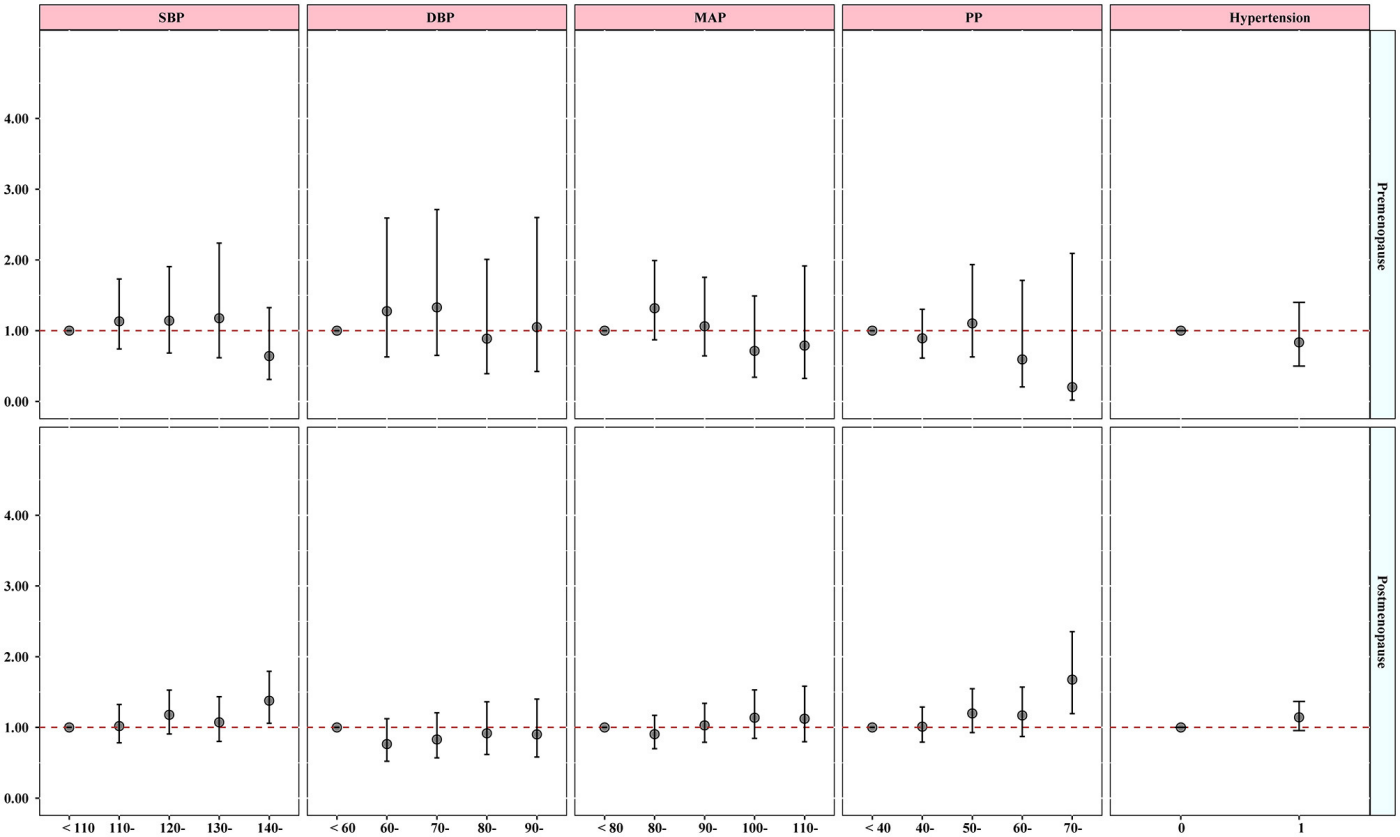
Association between blood pressure indicators and hypertension with osteoporosis considering menopause status. The model was adjusted by age, educational level, marital status, average monthly income, smoking, drinking status, physical activity, high-fat diet, increased vegetable and fruit intake, and BMI.

The results of sensitivity analyses are presented in Figure 3. The study found positive associations between SBP and PP, with osteoporosis only among postmenopausal women. The odds ratios (95% confidence intervals) were 1.02 (0.78, 1.33), 1.18 (0.91, 1.53), 1.07 (0.80, 1.44), and 1.38 (1.06, 1.79) for SBP and 1.01 (0.79, 1.29), 1.20 (0.93, 1.55), 1.17 (0.87, 1.57), and 1.68 (1.19, 2.35) for PP. The findings were supported by the dose–response relationships (see Supplementary Figure 5) and by excluding subjects who were taking medication (see Supplementary Figures 6, 7).

## Discussion

This cross-sectional study focuses on people with limited resources and aims to explore the association between different blood pressure proxies and osteoporosis. It is the first study of its kind, and it seeks to detect potential gender-difference associations. The results show that with increase in SBP, MAP, and PP, the prevalence of osteoporosis increases among women. Positive associations between SBP and PP with the risk of osteoporosis were observed in women only. In sensitivity analysis, similar conclusions were observed in postmenopausal women merely. The results were still robust even after excluding subjects taking medications, which suggests that the gender–difference association between blood pressure indicators and osteoporosis might be due to menopause status.

The prevalence of osteoporosis in China varies among studies. However, women are consistently more susceptible to the condition (3). A recent study conducted in China found that the occurrence of osteoporosis is more prevalent among older women compared to older men. Specifically, in northern China, the prevalence of osteoporosis was 36.9% among older women and 19.8% among older men. In contrast, in northwestern China, the prevalence was 9.65% among older women and 8.08% among older men (19). According to a nationwide study, osteoporosis affected 6.46% of men and 29.13% of women (4). According to a recent study, 29.0% of women and 13.5% of men suffer from osteoporosis on a national level (2), whereas in this research, the prevalence of osteoporosis was 14.07% for men and 22.64% for women in Chinese rural areas. In rural areas of China, men were the primary workforce and engaged in agricultural work, while women typically stayed at home as housewives (20). This resulted in men having not only a higher socioeconomic status but also being exposed to sunlight for longer periods of time than women. Additionally, previous research has found that Asian women tend to avoid sunlight exposure and skin-tanning, resulting in shorter sunlight exposure and lower levels of serum 25 (OH) D compared to men (21). Therefore, the prevalence of osteoporosis was higher in women than in men.

Numerous research studies have repeatedly linked hypertension with prevalent osteoporosis (22). An early cohort study conducted in 3,676 older adults white women found that increasing SBP corresponded with an increasing rate of bone loss (*P*_non − linear_ < 0.05) (9), which was similar to our results. A recent cross-section study also indicated that both SBP and DBP were inversely related with the BMD of proximal femoral and lumbar vertebral and that the beta values were −0.382, −0.290, and −0.340 of SBP and −0.318, −0.340, and −0.304 of DBP (23). A similar association was also observed in Chinese population (24, 25). Conducted in Tibet, a retrospective cross-sectional study also found that SBP was inversely associated with BMD T score of the spine and femoral neck or hip among diabetic postmenopausal women (24). A recent case-control study also indicated that hypertension was positively associated with osteoporosis (26). In contrast to our results, Javed et al. (27) reported that hypertension was not correlated with low BMD at either lumbar spine or both femoral necks among African American women aged over 65 years. A retrospective analysis also pronounced that there was no significant difference between hypertension and non-hypertension participants in the BMD of the femur or the spine (28). Evidence from Korea National Health and Nutrition indicated that lumbar spine osteoporosis was not significantly associated with blood pressure (29). A population-based Mendelian randomization study conducted among European populations also revealed a potential positive association between the PP and forearm BMD (30). These differences may be the results of different regions, environmental exposures, lifestyles, races, and other underlying factors.

The gender–difference association of between blood pressure with osteoporosis was repeatedly observed in previous research. For instance, Loke et al. (25) observed that both SBP and DBP were negatively associated with BMD among women but not significantly associated among men. A recent longitudinal study also highlighted that the BMD of the femoral neck was lower in women with hypertension than those without hypertension (0.80 vs. 0.82), while in men, hypertension was positively associated with the BMD of the lumbar spine and femoral neck (12). Additionally, evidence from a meta-analysis pronounced that the association between fracture and hypertension was slightly stronger in women (pooled *OR* = 1.52, 95% CI 1.30–1.79) than in men (pooled *OR* = 1.35, 95% CI 1.26–1.44) (11). Given the positive association between blood pressure and osteoporosis disappeared in premenopausal women, the gender–difference association may be attributed to the menopause status. Additionally, a lower sample size in men may also contribute to this statistical insignificance.

Despite the potential mechanism of blood pressure and osteoporosis not yet being clarified, limited studies still provided various pieces of evidence. Calcium may be a primary bridge between blood pressure and osteoporosis (31). Previous research has reported that participants with hypertension had a higher calcium elimination and a lower intestinal absorption than non-hypertension participants, which contributed to a lower calcium concentration in the plasma (31, 32). To sustain a suitable blood calcium level, bones may break down and release calcium into the blood (33). Therefore, the bone may be porous and prone to fractures (34). In addition, recent research also found that hypertension corresponded with the low level of 25-hydroxy vitamin D and osteocalcin, which led to a low bone turnover (10). Recent studies have found that angiotensin receptor blockers, selective beta-adrenergic receptor blockers, and thiazide diuretics may improve bone trabecular number and bone density by stimulating osteoblast differentiation and reducing osteoclast generation (35, 36), which supported that the medications of hypertension might impact the association.

During menopause and postmenopause, the reduced estrogen level in women would contribute to an increased osteoclastic resorption activity without a suitable increase in osteoblastic activity, which leads to a net loss of bone and a decreased bone strength (37–39). Thus, the low bone strength may explain that the significant association between blood pressure and osteoporosis was only observed among postmenopausal women but not among men or premenopausal women.

To the best of our knowledge, this is the first research to explore the gender–difference associations between divergent blood pressure proxies and osteoporosis among rural population. Despite that a large sample size and appropriate statistical methods could make this research more convincing, some limitations should be noted. First, only 7,689 participants from the Henan Rural Cohort study were included in this research and exclusion of participants with diseases and missing information may induce inevitable errors. Second, quantitative ultrasound (QUS) measures rather than X-ray absorptiometry (DXA) might underestimate osteoporosis prevalence; however, previous studies found it appeared capable of replacing dual X-ray considering its portability and low cost in the population-based study (40). Third, we did not examine the impact of vitamin D, calcium intake, and use of menopausal hormonal therapy and other medications on bone health, osteoprotegerin, and osteocalcin levels of the subjects, which may increase inevitable biases. Moreover, the unraveling reverse causality cannot be ruled out because of the survey based on a cross-sectional study. Finally, the results were based on the observations in rural populations, so caution is required when applying to other populations.

## Conclusion

While this study found a positive association between higher blood pressure (systolic and pulse) and osteoporosis in women living in resource-limited areas, it is crucial to note that this association was only significant for postmenopausal women. This suggests menopause itself may be a key factor in the observed gender difference. Therefore, while these findings raise the possibility of using blood pressure as a screening tool for osteoporosis in postmenopausal women with limited resources, further research is necessary to determine the generalizability of this association to all women, especially those yet to experience menopause.

## Data availability statement

The raw data supporting the conclusions of this article will be made available by the authors, without undue reservation.

## Ethics statement

The studies involving humans were approved by the Zhengzhou University Life Science Ethics Committee. The studies were conducted in accordance with the local legislation and institutional requirements. The participants provided their written informed consent to participate in this study.

## Author contributions

HJ: Formal analysis, Investigation, Methodology, Writing – original draft. HZ: Formal analysis, Investigation, Writing – original draft. SJ: Formal analysis, Investigation, Writing – review & editing. XY: Investigation, Writing – review & editing. XL: Data curation, Investigation, Writing – review & editing. CW: Data curation, Funding acquisition, Project administration, Supervision, Writing – review & editing. GZ: Investigation, Validation, Writing – review & editing. JP: Methodology, Writing – review & editing.
