# Supplementary material for: Menopause modified the association of blood pressure with osteoporosis among gender: a large-scale cross-sectional study
Source: Front Public Health. 2024 May 2;12:1383349. doi: 10.3389/fpubh.2024.1383349 (PMC11097953; doi:10.3389/fpubh.2024.1383349)
Supplement: Supplementary file 1 [file Data_Sheet_1.docx]

**Supplementary appendix**

**Supplement to:** Menopause modified the association of blood pressure with osteoporosis among sex: a large-scale cross-sectional study

**Supplementary table 1**. Baseline characteristics of participants by OP

| Variables | Non-OP | OP | *P* |
| --- | --- | --- | --- |
|  | n=6195 | n=1494 |  |
| Age, n (%) |  |  | <0.001 |
| 18- | 1026 (16.56) | 111 (7.43) |  |
| 45- | 4078 (65.83) | 811 (54.28) |  |
| 65- | 1091 (17.61) | 572 (38.29) |  |
| Gender, n (%) |  |  | <0.001 |
| Men | 2474 (39.94) | 405 (27.11) |  |
| Women | 3721 (60.06) | 1089 (72.89) |  |
| Educational level, n (%) |  |  | <0.001 |
| Elementary school or below | 2509 (40.50) | 868 (58.10) |  |
| Junior high school | 2712 (43.78) | 469 (31.39) |  |
| High school or above | 974 (15.72) | 157 (10.51) |  |
| Marital status, n (%) |  |  | <0.001 |
| Married/cohabitating | 5735 (92.57) | 1276 (85.41) |  |
| Unmarried/divorced /widowed | 460 (7.43) | 218 (14.59) |  |
| Average monthly income, n (%) |  |  | <0.001 |
| <500 RMB | 1891 (30.52) | 553 (37.01) |  |
| 500-1000 RMB | 1879 (30.33) | 444 (29.72) |  |
| ≥1000 RMB | 2425 (39.15) | 497 (33.27) |  |
| Smoking status, n (%) |  |  | <0.001 |
| Never | 4496 (73.54) | 1193 (80.55) |  |
| Former | 324 (5.30) | 40 (2.70) |  |
| Current | 1294 (21.16) | 248 (16.75) |  |
| Drinking status, n (%) |  |  | <0.001 |
| Never | 4979 (76.50) | 1239 (83.66) |  |
| Former | 929 (15.19) | 148 (9.99) |  |
| Current | 508 (8.31) | 94 (6.35) |  |
| Physical activity, n (%) |  |  | 0.431 |
| Low | 1716 (27.70) | 435 (29.12) |  |
| Moderate | 2405 (38.82) | 556 (37.22) |  |
| High | 2074 (39.70) | 503 (33.67) |  |
| High-fat diet, n (%) | 1354 (33.48) | 198 (13.25) | <0.001 |
| More vegetable and fruit intake, n (%) | 3414 (55.11) | 646 (43.24) | <0.001 |
| Hypertension, n (%) | 1490 (24.05) | 442 (29.59) | <0.001 |
| Postmenopausal women, n (%) | 2130 (34.51) | 921 (61.85) | <0.001 |
| BMI (kg/m^2^, mean ± SD) | 24.84±3.37 | 23.86±3.50 | <0.001 |
| SBP (mmHg, mean ± SD) | 121.36±18.04 | 124.97±19.77 | <0.001 |
| DBP (mmHg, mean ± SD) | 75.69±11.00 | 74.99±11.32 | 0.030 |
| MAP (mmHg, mean ± SD) | 90.91±12.65 | 91.65±13.27 | 0.050 |
| PP (mmHg, mean ± SD) | 45.68±11.43 | 49.98±13.34 | <0.001 |

Abbreviations: BMI: body mass index; SBP: systolic blood pressure; DBP: diastolic blood pressure; MAP: mean arterial pressure; PP: pulse pressure; OP: osteoporosis.

**Supplementary table 2**. Baseline characteristics of participants by HTN

| Variables | | | Non-HTN | HTN | *P* |
| --- | --- | --- | --- | --- | --- |
|  |  |  | n=5757 | n=1932 |  |
| Age, n (%) | | |  |  | <0.001 |
| 18- | | | 1019 (17.70) | 118 (16.05) |  |
| 45- | | | 3682 (63.96) | 1207 (64.93) |  |
| 65- | | | 1056 (18.34) | 915 (31.42) |  |
| Gender | | |  |  | 0.110 |
| Men | | | 2185 (37.95) | 694 (35.92) |  |
| Women | | | 3572 (62.05) | 1238 (64.09) |  |
| Educational level, n (%) | | |  |  | <0.001 |
| Elementary school or below | | | 2420 (42.03) | 957 (49.53) |  |
| Junior high school | | | 2472 (42.94) | 709 (36.70) |  |
| High school or above | | | 865 (15.03) | 266 (13.77) |  |
| Marital status, n (%) | | |  |  | 0.138 |
| Married/cohabitating | | | 5290 (91.89) | 1721 (89.08) |  |
| Unmarried/divorced /widowed | | | 467 (8.11) | 211 (10.92) |  |
| Average monthly income, n (%) |  |  |  |  |  |
| <500 RMB | | | 1822 (31.65) | 622 (32.19) |  |
| 500-1000 RMB | | | 1726 (29.98) | 597 (30.90) |  |
| ≥1000 RMB | | | 2209 (38.37) | 713 (36.91) |  |
| Smoking status, n (%) | | |  |  | 0.071 |
| Never | | | 4217 (74.24) | 1472 (76.87) |  |
| Former | | | 278 (4.89) | 86 (4.49) |  |
| Current | | | 1185 (20.87) | 357 (18.64) |  |
| Drinking status, n (%) | | |  |  | 0.016 |
| Never | | | 4407 (77.56) | 1511 (78.90) |  |
| Former | | | 840 (14.78) | 237 (12.38) |  |
| Current | | | 435 (7.66) | 167 (8.72) |  |
| Physical activity, n (%) | | |  |  | 0.065 |
| Low | | | 1573 (27.32) | 578 (29.92) |  |
| Moderate | | | 2250 (39.08) | 711 (36.80) |  |
| High | | | 1934 (33.59) | 643 (33.28) |  |
| High-fat diet, n (%) | | | 783 (21.02) | 769 (17.70) | 0.002 |
| More vegetable and fruit intake, n (%) | | | 3122 (54.23) | 938 (48.55) | <0.001 |
| Osteoporosis, n (%) | | | 1052 (18.27) | 422 (22.88) | 0.110 |
| Postmenopausal women, n (%) | | | 2070 (36.11) | 981 (50.88) | <0.001 |
| BMI (kg/m^2^, mean ± SD) | | | 24.21±3.29 | 25.96±3.47 | <0.001 |
| SBP (mmHg, mean ± SD) | | | 114.50±11.79 | 144.61±16.15 | <0.001 |
| DBP (mmHg, mean ± SD) | | | 71.68±8.12 | 87.09±10.64 | <0.001 |
| MAP (mmHg, mean ± SD) | | | 85.95±8.59 | 106.26±11.01 | <0.001 |
| PP (mmHg, mean ± SD) | | | 42.82±8.62 | 57.52±13.61 | <0.001 |

Abbreviations: BMI: body mass index; SBP: systolic blood pressure; DBP: diastolic blood pressure; MAP: mean arterial pressure; PP: pulse pressure; HTN: hypertension.

**Supplementary table 3**. Relationship between blood pressure indicators and the risk of osteoporosis in the elderly by gender (≥50 years old)

| Variables |  | Men (n=2167)  *OR* (95%*CI*) | Women (n=3391) |
| --- | --- | --- | --- |
|  |  |  | *OR* (95%*CI*) |
| SBP | |  |  |
| < 110 | | 1.00 | 1.00 |
| 110- | | 0.99 (0.70, 1.40) | 1.08 (0.84, 1.39) |
| 120- | | 0.83 (0.56, 1.22) | **1.40 (1.09, 1.80)** |
| 130- | | 0.95 (0.62, 1.47) | **1.35 (1.02, 1.78)** |
| 140- | | 1.37 (0.94, 1.98) | **1.66 (1.29, 2.12)** |
| DBP | |  |  |
| < 60 | | 1.00 | 1.00 |
| 60- | | 1.54 (0.84, 2.84) | 0.74 (0.51, 1.06) |
| 70- | | 1.45 (0.79, 2.68) | 0.77 (0.54, 1.10) |
| 80- | | 1.21 (0.62, 2.32) | 0.83 (0.57, 1.21) |
| 90- | | **2.62 (1.34, 5.14)** | 0.80 (0.52, 1.21) |
| MAP | |  |  |
| <80 | | 1.00 | 1.00 |
| 80- | | 0.97 (0.68, 1.38) | 0.94 (0.73, 1.20) |
| 90- | | 0.91 (0.62, 1.34) | 1.11 (0.86, 1.43) |
| 100- | | 0.92 (0.59, 1.44) | 1.20 (0.90, 1.60) |
| 110- | | **1.81 (1.14, 3.87)** | 1.21 (0.87, 1.68) |
| PP | |  |  |
| <40 | | 1.00 | 1.00 |
| 40- | | 0.98 (0.71, 1.34) | 1.15 (0.91, 1.46) |
| 50- | | 1.06 (0.74, 1.51) | **1.53 (1.20, 1.95)** |
| 60- | | 1.18 (0.74, 1.89) | **1.55 (1.18, 2.05)** |
| 70- | | 1.42 (0.82, 2.47) | **2.49 (1.81, 3.44)** |

Abbreviations: BMI: body mass index; SBP: systolic blood pressure; DBP: diastolic blood pressure; MAP: mean arterial pressure; PP: pulse pressure.

Bold font: *P* <0.05.

Adjustment for educational level, marital status, average monthly income, smoking, drinking status, physical activity, high-fat diet, more vegetable and fruit intake, and BMI.

**Figure Legends**

**Supplementary Figure 1:** This figure compares the prevalence of osteoporosis across different blood pressure indicator groups (SBP, DBP, MAP, PP), analyzed separately for men and women. Participants taking medications are excluded from the analysis.

**Supplementary Figure 2:** The dose-response relationship between blood pressure and osteoporosis. The model adjusts for potential confounding factors, including age, gender, education level, marital status, income, smoking, drinking, physical activity, higt fat diet, more vegetable and fruit intake, and BMI.

**Supplementary Figure 3:** This figure analyzes the association between blood pressure indicators (SBP, DBP, MAP, PP), hypertension status, and osteoporosis, focusing on gender differences. Participants taking medications are excluded from the analysis. The model is adjusted for age, education level, marital status, income, smoking, drinking, physical activity, high fat diet, more vegetable and fruit intake, and BMI.

**Supplementary Figure 4:** This figure explores the dose-response relationship between blood pressure and osteoporosis, excluding participants taking medications. The analysis is adjusted for age, gender, education level, marital status, income, smoking, drinking, physical activity, high fat diet, more vegetable and fruit intake, and BMI.

**Supplementary Figure 5:** This figure explores the dose-response relationship between blood pressure indicators (SBP, DBP, MAP, PP) and osteoporosis prevalence, stratified by menopausal status. The model is adjusted for age, education level, marital status, income, smoking, drinking, physical activity, high fat diet, more vegetable and fruit intake, and BMI.

**Supplementary Figure 6:** This figure analyzes the association between blood pressure indicators (SBP, DBP, MAP, PP), hypertension status, and osteoporosis prevalence, stratified by menopausal status. Participants taking medications are excluded from the analysis. The model is adjusted for age, education level, marital status, income, smoking, drinking, physical activity, high fat diet, more vegetable and fruit intake, and BMI.

**Supplementary Figure 7:** This figure explores the dose-response relationship between blood pressure indicators (SBP, DBP, MAP, PP) and osteoporosis, stratified by menopause status. Participants taking medications are excluded from the analysis. The model is adjusted for age, education level, marital status, income, smoking, drinking, physical activity, high fat diet, more vegetable and fruit intake, and BMI.


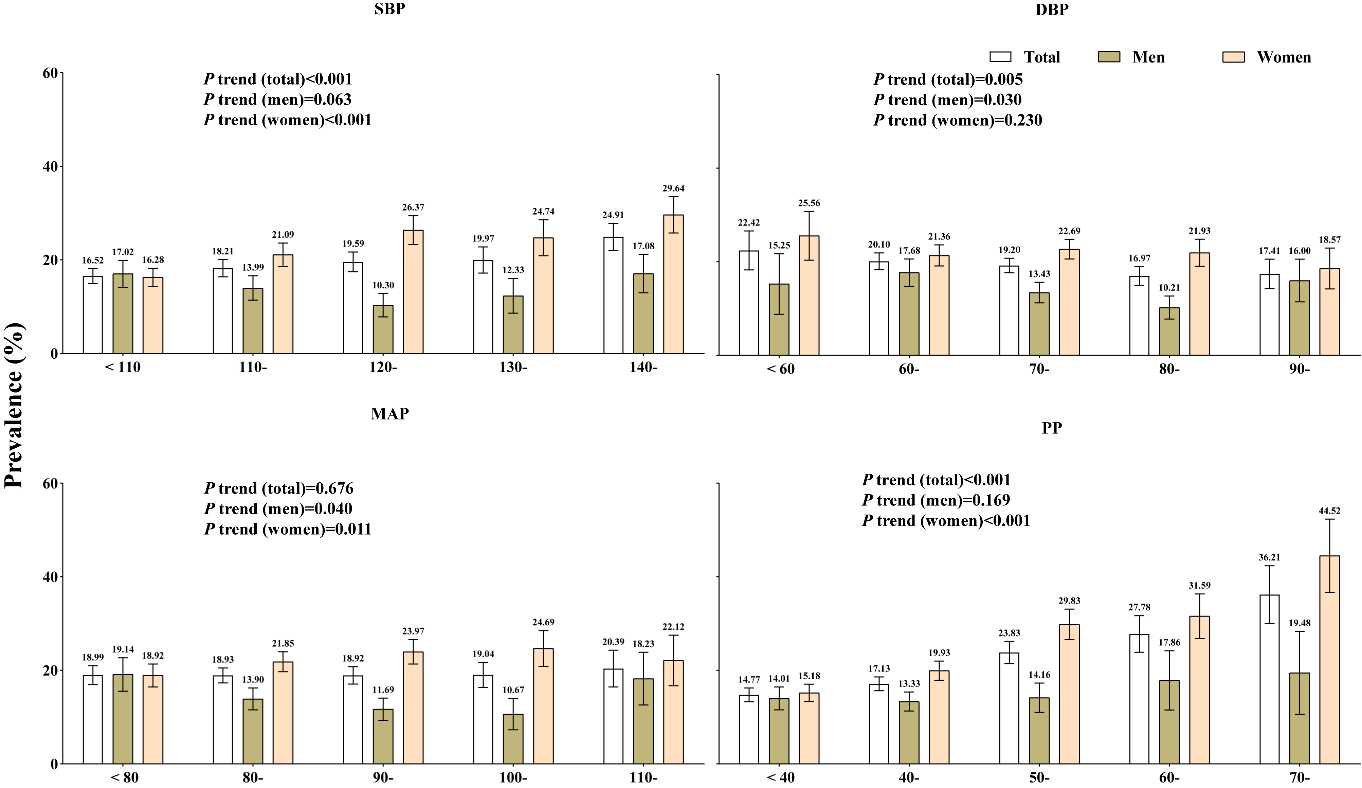


**Figure 1 (Supplementary):** This figure compares the prevalence of osteoporosis across different blood pressure indicator groups (SBP, DBP, MAP, PP), analyzed separately for men and women. Participants taking medications are excluded from the analysis.


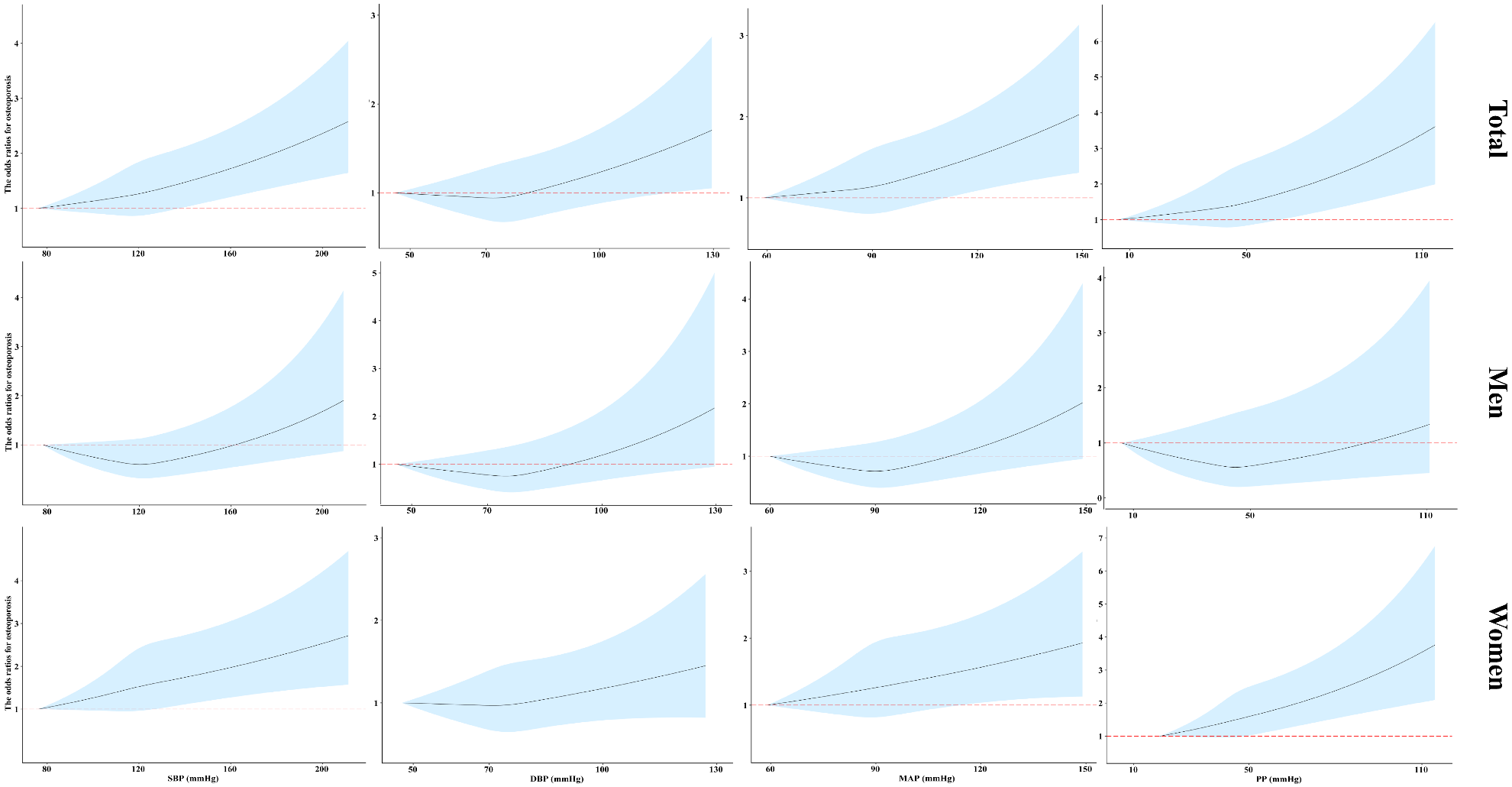


**Figure 2 (Supplementary)** illustrates the dose-response relationship between blood pressure and osteoporosis. The model adjusts for potential confounding factors, including age, gender, education level, marital status, income, smoking, drinking, physical activity, high-fat diet, fruit and vegetable intake, and BMI.
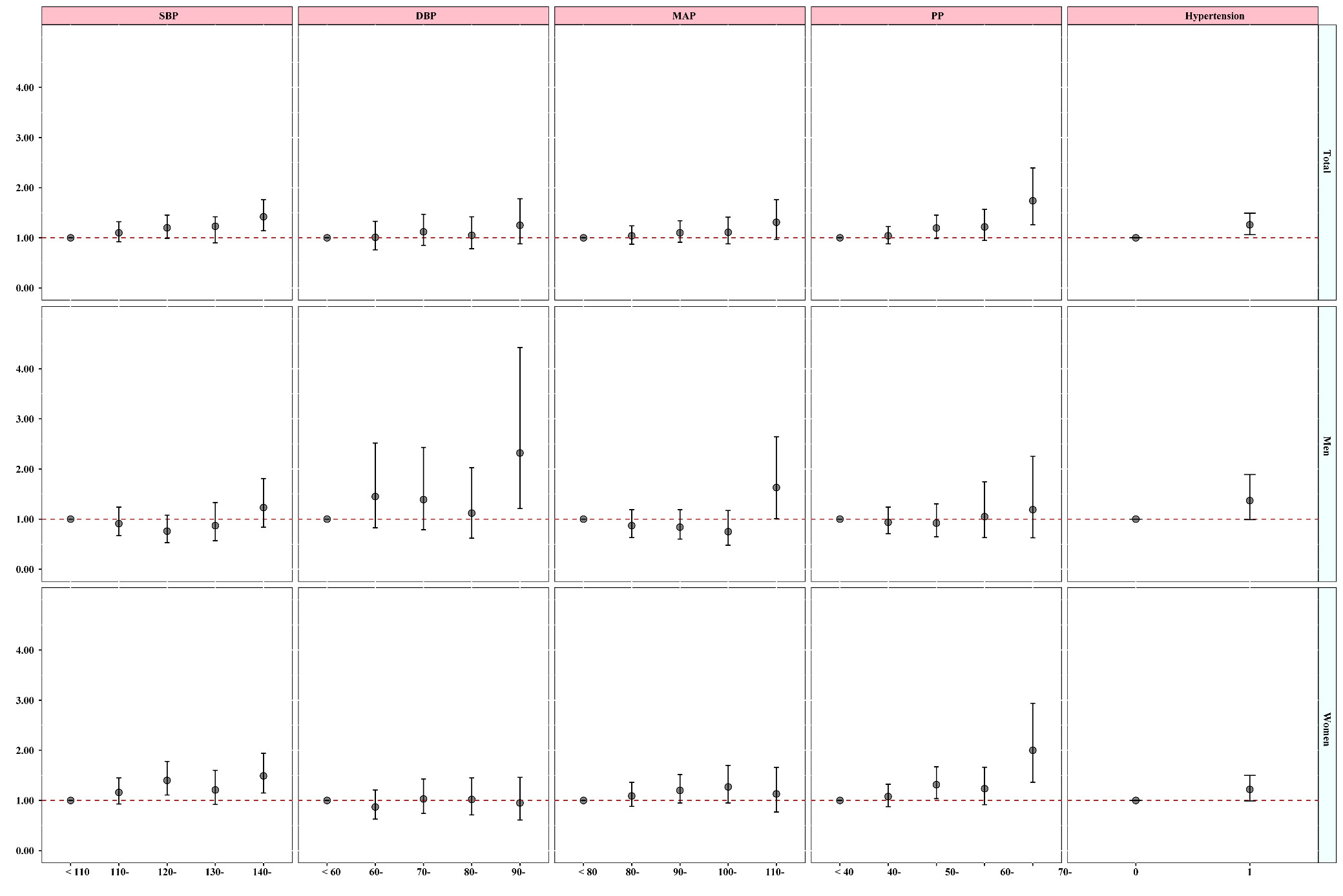
 **Figure 3 (Supplementary):** This figure analyzes the association between blood pressure indicators (SBP, DBP, MAP, PP), hypertension status, and osteoporosis, focusing on gender differences. Participants taking medications are excluded from the analysis. The model is adjusted for age, education level, marital status, income, smoking, drinking, physical activity, high fat diet, fruit and vegetable intake, and BMI.


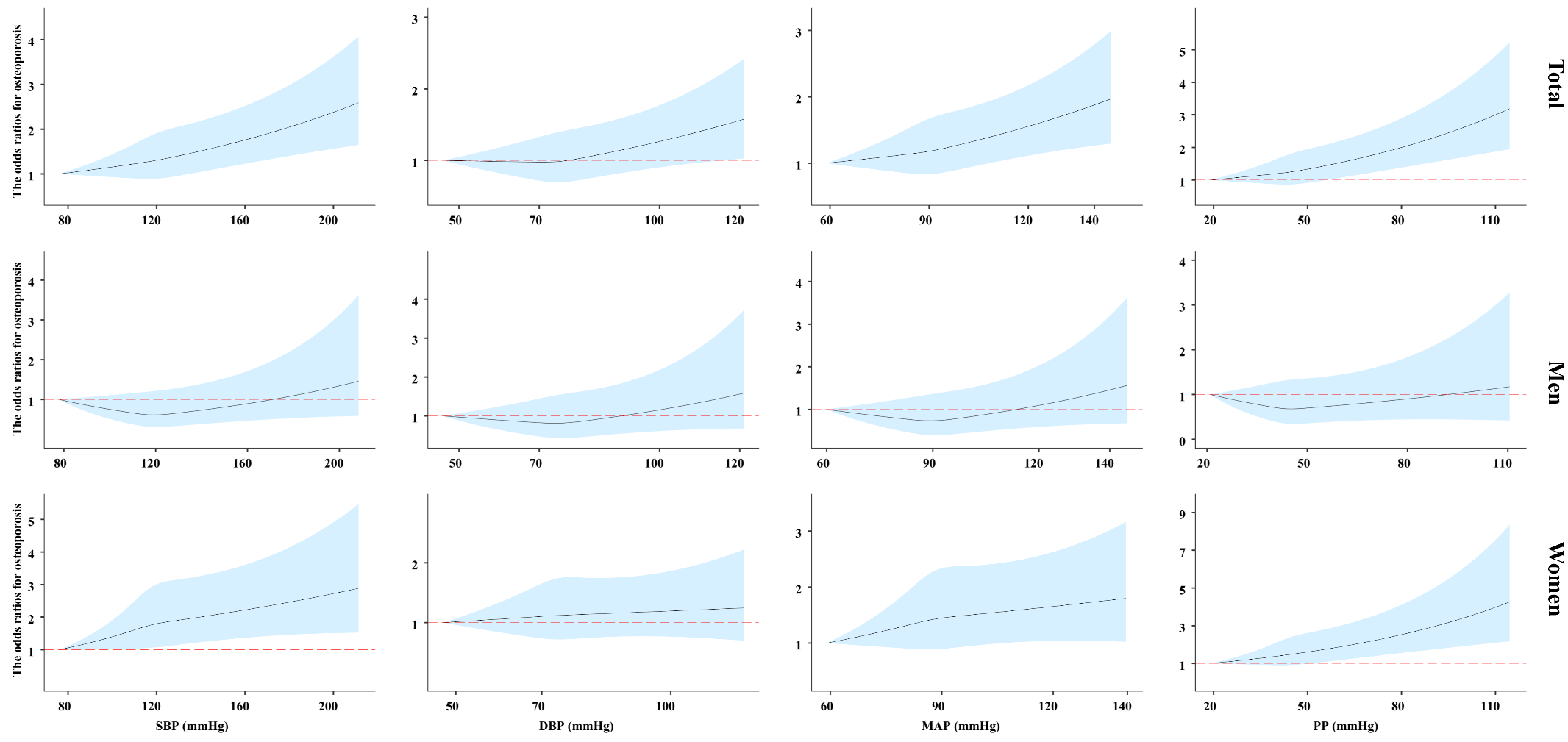


**Figure 4 (Supplementary):** This figure explores the dose-response relationship between blood pressure and osteoporosis, excluding participants taking medications. The analysis is adjusted for age, gender, education level, marital status, income, smoking, drinking, physical activity, high fat diet, more vegetable and fruit intake, and BMI.


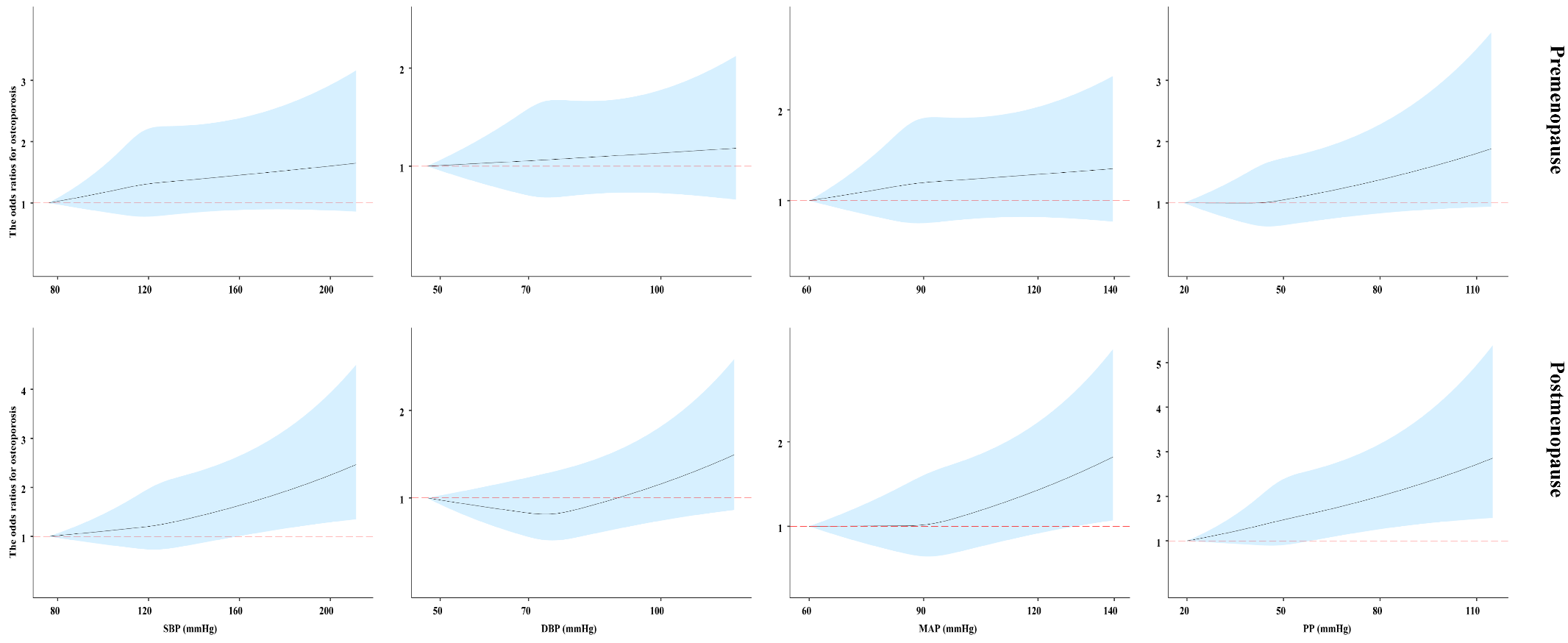
**Figure 5 (Supplementary):** This figure explores the dose-response relationship between blood pressure indicators (SBP, DBP, MAP, PP) and osteoporosis prevalence, stratified by menopausal status. The model is adjusted for age, education level, marital status, income, smoking, drinking, physical activity, high fat diet, more vegetable and fruit intake, and BMI.


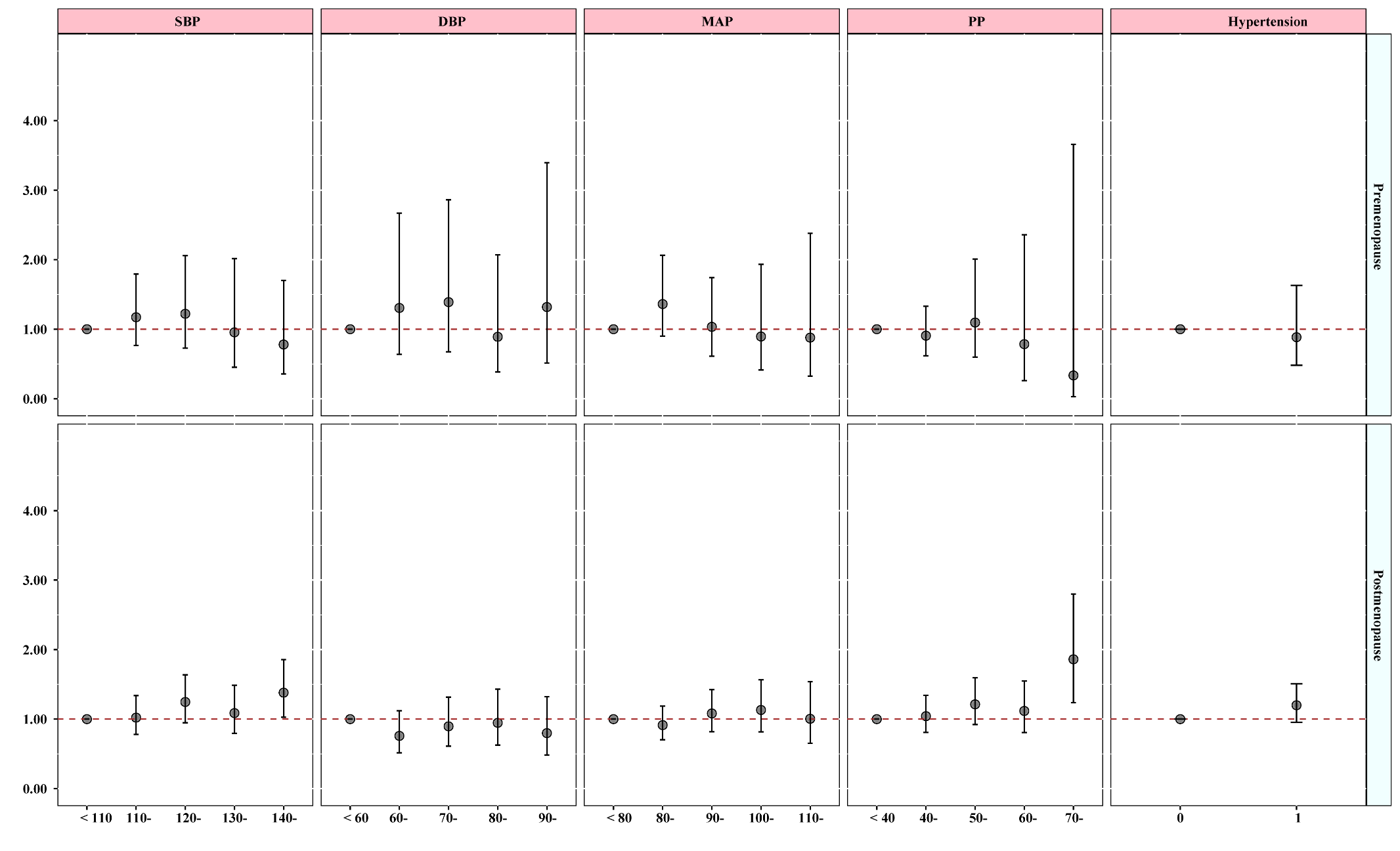
 **Figure 6 (Supplementary):** This figure analyzes the association between blood pressure indicators (SBP, DBP, MAP, PP), hypertension status, and osteoporosis prevalence, stratified by menopausal status. Participants taking medications are excluded from the analysis. The model is adjusted for age, education level, marital status, income, smoking, drinking, physical activity, high fat diet, more vegetable and fruit intake, and BMI.


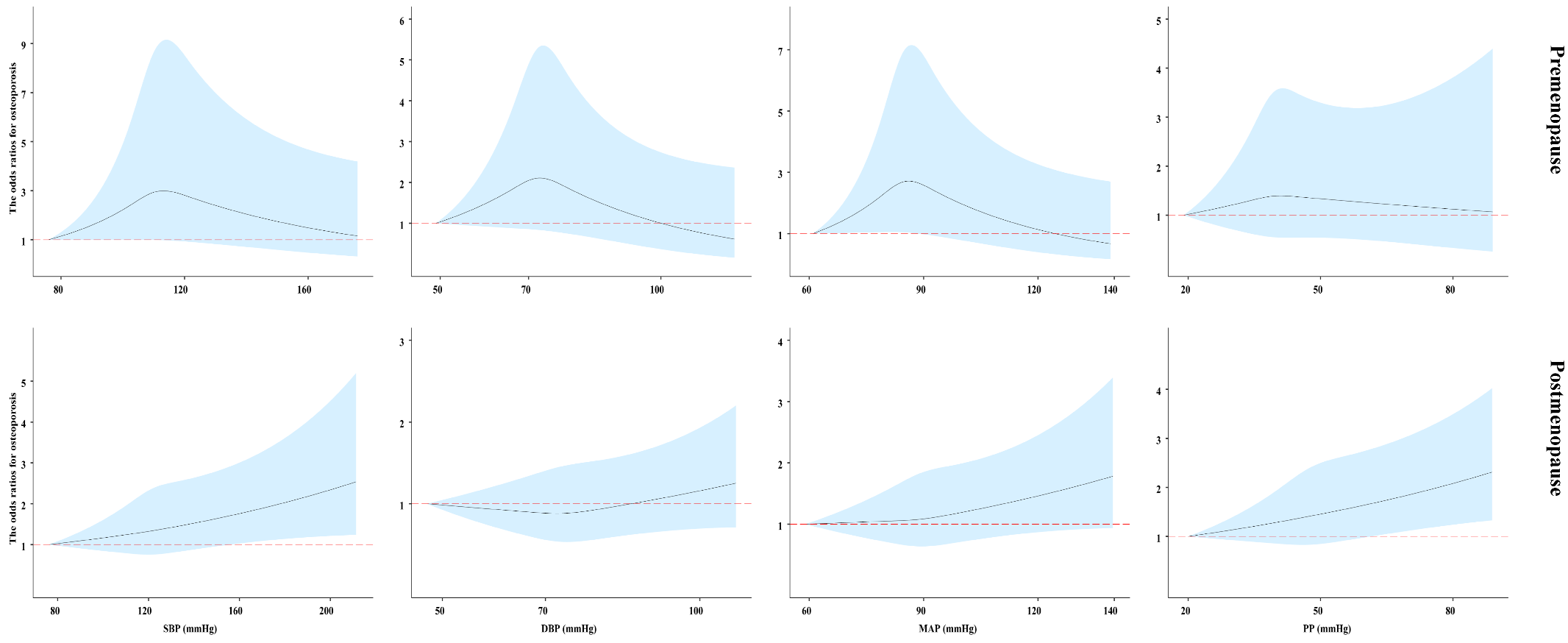


**Figure 7 (Supplementary):** This figure explores the dose-response relationship between blood pressure indicators (SBP, DBP, MAP, PP) and osteoporosis, stratified by menopause status. Participants taking medications are excluded from the analysis. The model is adjusted for age, education level, marital status, income, smoking, drinking, physical activity, high fat diet, more vegetable and fruit intake, and BMI.
